# Evaluation of functional and metabolic tumor volume using voxel-wise analysis in childhood rhabdomyosarcoma

**DOI:** 10.1007/s00247-022-05540-2

**Published:** 2022-11-18

**Authors:** Simon Maennlin, Maryanna Chaika, Sebastian Gassenmaier, Robert Grimm, Monika Sparber-Sauer, Jörg Fuchs, Andreas Schmidt, Martin Ebinger, Simone Hettmer, Sergios Gatidids, Helmut Dittmann, Jürgen F. Schäfer

**Affiliations:** 1 Department of Diagnostic and Interventional Radiology, University Hospital Tuebingen, Hoppe-Seyler-Strasse 3, 72076 Tuebingen, Germany; 2 MR Application Predevelopment, Siemens Healthcare GmbH, Erlangen, Germany; 3 Pediatrics 5, Klinikum Stuttgart, Olgahospital, Stuttgart, Germany; 4 Department of Pediatric Surgery and Pediatric Urology, University Children’s Hospital Tuebingen, Tuebingen, Germany; 5 Department of Hematology and Oncology, University Children’s Hospital Tübingen, Tuebingen, Germany; 6 Department of Pediatrics and Adolescent Medicine, Division of Pediatric Hematology and Oncology, Faculty of Medicine, University of Freiburg, Freiburg, Germany; 7 Department of Nuclear Medicine, University Hospital Tuebingen, Tuebingen, Germany

**Keywords:** Children, Computed tomography, Diffusion-weighted imaging, Magnetic resonance imaging, Multimodal imaging, Positron emission tomography, Prognosis, Rhabdomyosarcoma

## Abstract

**Background:**

Cross-sectional imaging-based morphological characteristics of pediatric rhabdomyosarcoma have failed to predict outcomes.

**Objective:**

To evaluate the feasibility and possible value of generating tumor sub-volumes using voxel-wise analysis of metabolic and functional data from positron emission tomography/magnetic resonance imaging (PET/MR) or PET/computed tomography (CT) and MRI in rhabdomyosarcoma.

**Materials and methods:**

Thirty-four examinations in 17 patients who received PET/MRI or PET/CT plus MRI were analyzed. The volume of interest included total tumor volume before and after therapy. Apparent diffusion coefficients (ADC) and standard uptake values (SUV) were determined voxel-wise. Voxels were assigned to three different groups based on ADC and SUV: “viable tumor tissue,” “intermediate tissue” or “possible necrosis.” In a second approach, data were grouped into three clusters using the Gaussian mixture model. The ratio of these clusters to total tumor volume and changes due to chemotherapy were correlated with clinical and histopathological data.

**Results:**

After chemotherapy, the proportion of voxels in the different groups changed significantly. A significant reduction of the proportion of voxels assigned to cluster 1 was found, from a mean of 36.4% to 2.5% (*P* < 0.001). There was a significant increase in the proportion of voxels in cluster 3 following chemotherapy from 24.8% to 81.6% (*P* = 0.02). The proportion of voxels in cluster 2 differed depending on the presence or absence of tumor recurrence, falling from 48% to 10% post-chemotherapy in the group with no tumor recurrence (*P* < 0.05) and from 29% to 23% (*P* > 0.05) in the group with tumor recurrence.

**Conclusion:**

Voxel-wise evaluation of multimodal data in rhabdomyosarcoma is feasible. Our initial results suggest that the different distribution of sub-volumes before and after therapy may have prognostic significance.

## Introduction

Rhabdomyosarcoma is the most common malignant soft-tissue tumor in children, adolescents and young adults with an annual incidence of 1:170,000 [1, 2]. Generally, two histological subtypes, embryonal and alveolar, are differentiated [1]. The alveolar subtype is associated with a poorer response to therapy and a reduced prognosis [3]. The 5-year survival rate of rhabdomyosarcoma remains unsatisfactory despite modern therapy protocols: 75% in localized disease, 30% in metastatic disease [4, 5].

Established prognostic biomarkers for patients with rhabdomyosarcoma that aim to predict therapy success and clinical outcome are age, localization, anatomical tumor size, histology, molecular marker, surgical resectability and metastases [1]. Morphological characteristics such as tumor size reduction after chemotherapy on cross-sectional imaging such as magnetic resonance imaging (MRI) and computed tomography (CT) have failed to predict clinical outcome in patients with rhabdomyosarcoma [6–8].

Some studies have investigated the significance of advanced imaging such as diffusion-weighted imaging (DWI) and 2-deoxy-2-[^18^F] fluoro-D-glucose (^18^F-FDG) positron emission tomography (PET). Metabolic uptake evaluated on ^18^F-FDG PET has prognostic value in pediatric rhabdomyosarcoma [9]. Additionally, it has been demonstrated that the degree and extent of restricted diffusion on DWI and the combination of hypermetabolism on ^18^F-FDG PET and restricted diffusion can predict outcomes in malignant disease [10, 11].

Therefore, the imaging committee of the European Pediatric Soft Tissue Sarcoma Study Group (EpSSG 2021) highlighted the potential of PET and MR, but still recommends MRI only for assessment of primary tumor due to a lack of evidence in support of PET [12].

Addressing the inhomogeneity of malignant tumors, recent studies demonstrate the possibility and advantage of analyzing tumor volumes on a voxel-wise basis rather than investigating whole or partial tumor volumes [13, 14].

As tumor tissue of rhabdomyosarcoma is also often observed to be heterogenous, we hypothesize that a voxel-wise analysis based on DWI and ^18^F-FDG PET values before and after chemotherapy might be suitable to characterize tumor sub-volumes.

## Materials and methods

### Patient selection

Patients were retrospectively selected from two databases: (1) from a prospectively collated PET/MRI registry study at the Department of Diagnostic and Interventional Radiology, University Hospital Tuebingen and (2) from the cooperative soft-tissue sarcoma group CWS-SoTiSaR. The studies were approved by the corresponding institutional review boards. Imaging was conducted between January 2015 and December 2020. Written consent was given by all adult patients and all parents of pediatric patients. Inclusion criteria were (a) younger than 18 years old, (b) histologically confirmed rhabdomyosarcoma, (c) PET/MRI or PET/CT and MRI including DWI performed within 7 days, (d) imaging performed before and after induction chemotherapy and (e) reasonable image quality of the primary tumor region without severe artifacts.

### Image acquisition

Patients recruited from the University Hospital Tuebingen registry underwent whole-body ^18^F-FDG PET/MRI performed on an integrated clinical PET/MRI system (Biograph mMR; Siemens Healthcare GmbH, Erlangen, Germany). Each patient was examined by acquiring a whole-body PET/MRI with simultaneous acquisition of PET and MRI. Patients were instructed to fast for at least 6 h before intravenous injection of ^18^F- FDG in preparation for their PET scan. The administered dose of ^18^F-FDG was calculated based on patient body weight (mean: 4.8 MBq) [15]. PET acquisition was executed approximately 60 min after tracer injection.

For PET attenuation correction, a segmentation-based map was generated by applying a standardized whole-body 3-D T1-weighted spoiled gradient echo sequence with Dixon-based fat-water separation [16]. Patients recruited from the CWS-SoTiSaR database underwent ^18^F-FDG PET/CT using a clinical PET/CT scanner and MRI within three days (Philips Gemini TF [Best, The Netherlands], Siemens Biograph mCT [Erlangen, Germany], Philips Achieva 1.5 T [Best, The Netherlands], Siemens Symphony 1.5 T [Best, The Netherlands]).

For both groups of patients, MR imaging included at least an axial T2-weighted sequence and DWI. The MRI protocol is shown in Table 1.

**Table 1:** Acquisition parameters used in our magnetic resonance imaging examination protocol

	T1 Dixon axial	T2 STIR coronal	T2 STIR axial	T2 TSE axial	DWI	T1 VIBE Dixon axial pKM
TE (ms)	1.23/2.46	78	81	100	60	1.29
TR (ms)	3.6	6,400	4,500	3,500	6,000	4.05
Bandwidth (Hz/px)	965	383	220	260	1,860	1,040
Matrix size	79 × 192	256 × 256	197 × 384	256 × 300	108 × 192	329/165
Resolution [mm^3^]	4.1 × 2.6x2.6	1.5 × 1.5x4	1.2 × 0.83x5	1.25 × 1.25x5	2.6 × 2.6x5	1.2x.1.2 × 3
Excitation angle [°]	10	120	120	90	90	10
Inversion time [ms]		200	220			
*b*-values [mm^2^/s]					50 and 800	

*DWI* diffusion-weighted image, *pKM* pharmacokinetics modelling, *STIR* short tau inversion recovery, *TE* echo time, *TR* repetition time, *VIBE* volumetric interpolated breath-hold examination, *TSE *turbo spin echo

### Data processing

A dedicated software prototype (Multiparametric Analysis Siemens Healthcare) was used for voxel-by-voxel analysis. T2-weighted images, apparent diffusion coefficient (ADC) maps and PET images were automatically fused and coregistered according to DICOM tag information using a non-rigid fusion function. Due to the different image resolution of PET images compared to ADC maps and T2-weighted images, the voxel size of these modalities was interpolated to 0.7 × 0.7 × 5 mm. Correlation of anatomy on the various coregistered modalities was visually confirmed by one radiologist with 2 years of experience in pediatric hybrid imaging (S.M.) and confirmed by a board-certified pediatric radiologist with 30 years of experience and sub-specialization in pediatric radiology (J.F.S.). Volumes of interest were drawn using the T2-weighted images, covering the whole primary tumor mass. All solid and necrotic parts of the primary tumor mass were enclosed. Major blood vessels were excluded.

Separately acquired data using PET/CT followed by MRI were fused and coregistered manually for CT images, PET images and ADC maps, focusing on the tumor region. Image fusion and registration were performed and confirmed by S.M and J.F.S., respectively. The voxel size of the CT images and ADC maps was adapted to the PET voxel size (4 × 4 × 4 mm for Philips Gemini and 4 × 4 × 5 mm for Siemens Biograph mCT). The volumes of interest were drawn based on axial CT images, including the total primary tumor volume.

### Data analysis

**Fig. 1:**
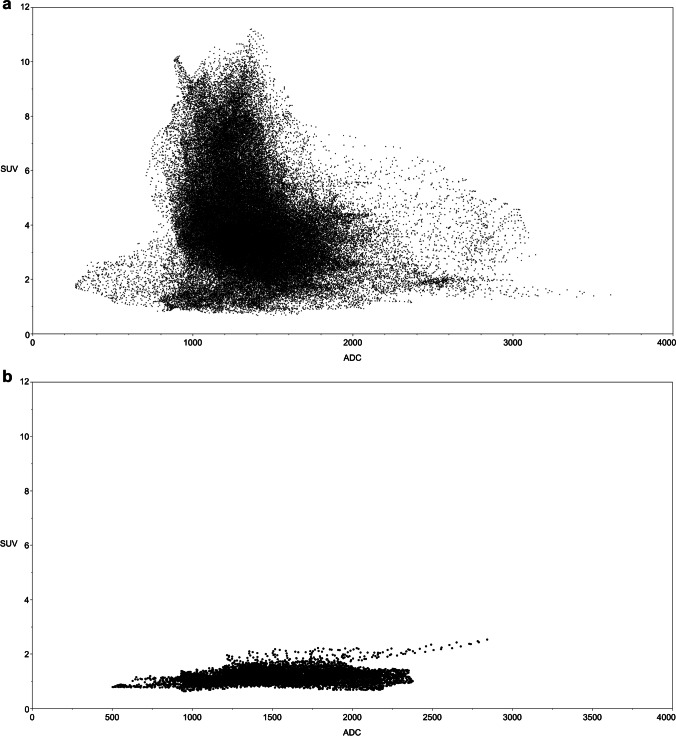
Scatterplot of a 62-month-old boy with pelvic embryonal rhabdomyosarcoma. Standard uptake value (SUV) and apparent diffusion coefficient (ADC) data were acquired using PET/MRI (**a**) pre- and (**b**) post-chemotherapy

The software prototype generated standard uptake values (SUV) and ADC for each voxel. Figure 1 shows an example scatterplot of the two variables. Data were exported to statistical Software (JMP Software version 16.1 from SAS).

As a first step, voxels were grouped into sub-volumes using threshold values for SUV and ADC: SUV = 2.5 and ADC = 1,000 × 10^–6^ mm^2^/s. The thresholds were chosen according to the literature for a rough estimation between malignant and benign tissue [17–20]. Accordingly, four groups of voxels resulted: (1) high SUV and low ADC, (2) high SUV and high ADC, (3) low SUV and low ADC and (4) low SUV and high ADC. Three sub-volumes were formed from these four groups: To the first sub-volume, named “viable tissue,” all voxels were assigned with SUV > 2.5 and ADC < 1,000 × 10^–6^ mm2/s. All voxels with SUV > 2.5 or ADC < 1,000 × 10^–6^ mm2/s were assigned to the second sub-volume, which was named “intermediate tissue.” If SUV ≤ 2.5 and ADC ≥ 1,000 × 10^–6^ mm2/s, voxels were assigned to the third group named “possible necrosis.”

### Gaussian mixture model clustering

The liver mean SUV (SUV_mean)_ was obtained for each data set by drawing a volume of interest of 14 ml within the liver tissue, avoiding large vessels or bile ducts. Voxel-by-voxel SUV values of analyzed tumor regions were normalized to liver SUV_mean_. Normalization to the liver value was performed to mitigate differences in SUV values between different sites and scanners. Each data set was reduced or extrapolated to the same volume of 1,000 voxels to minimize aberrance effects by interindividual variable tumor sizes. Obtained ADC and SUV values were normalized collectively using the following formula:

$$x_{norm}=\frac{x-mean(x)}{std\;dev(x)}$$

As reported in previous studies, a voxel-based approach for ^18^F-FDG PET and DWI was applied using the Gaussian mixture model [13, 21]. Therefore, three Gaussian distributions were fitted to the collective data and intersections generated by the two following Gaussian distributions were established as thresholds for the separation of voxel-by-voxel data into tumor sub-volumes.

**Fig. 2:**
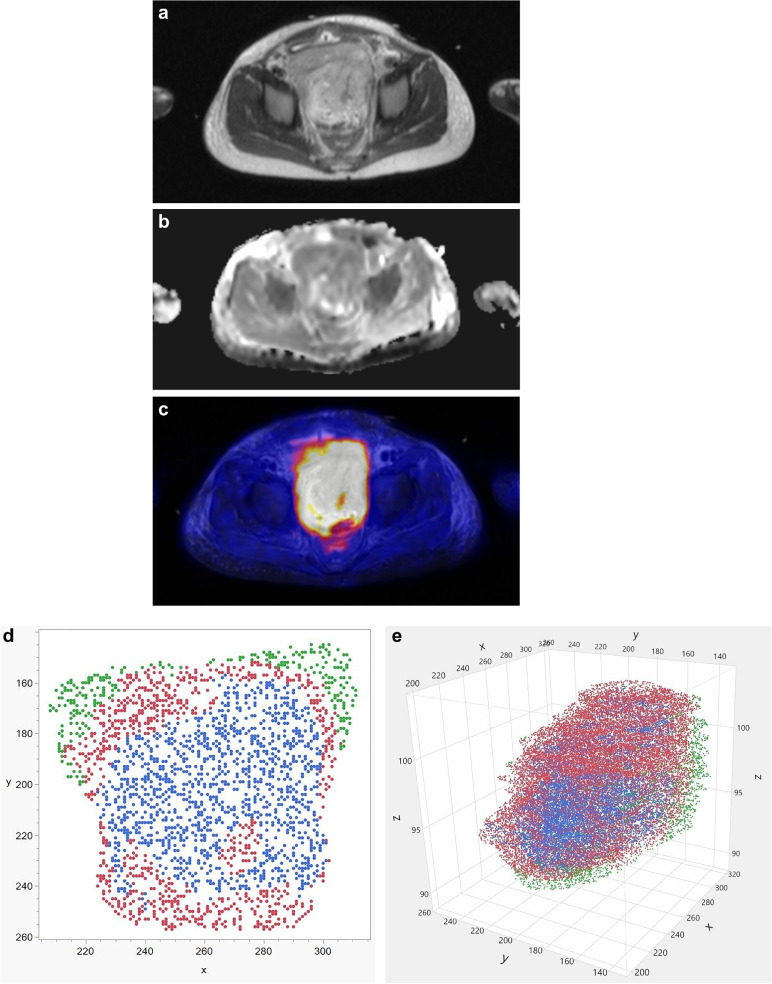
Overview of the clustering process analyzing a single slice from a PET/MRI data set in a 62-month-old boy with pelvic embryonal rhabdomyosarcoma before chemotherapy (same boy as in Fig. 1). **a** Axial T2-weighted image. **b** Axial apparent diffusion coefficient map. **c** Axial fused image of corrected PET data set and T2-weighted image. **d** After clustering, the voxels in the slice shown in (**a-c**) are distributed to one of three clusters: cluster 1 = red, cluster 2 = blue, cluster 3 = green. **e** Volume of interest encompassing the entire tumor volume. After clustering, voxels are distributed to one of three clusters: cluster 1 = red, cluster 2 = blue, cluster 3 = green

Tumor sub-volumes were compared before and after chemotherapy regarding mean SUV and ADC values, using a Wilcoxon signed-rank test. Changes in sub-volumes through therapy were correlated with clinical and histopathological patient data applying point-biserial correlation and linear regression. *P*-values < 0.05 were considered significant. Figure 2 gives an overview of the different steps involved in clustering a data set acquired by PET/MRI.

## Results

Table 2 shows the characteristics of the patients in this study. At the time of baseline imaging, the median age was 62 months (range: 7–196 months). Eleven patients underwent PET/MRI and six patients underwent PET/CT plus MRI. Most patients presented with a genitourinary primary tumor site (88%) and with embryonal subtype (71%). Resection of the primary tumor after chemotherapy was performed in 11 patients (64%). Six of the resected specimens showed histopathological viability. Recurrence occurred in 47% of patients. In one patient, a local event occurred; in five patients, a metastatic event occurred and in two patients both local and metastatic events occurred.

**Table 2:** Demographic and clinical characteristics of the 17 patients

Characteristic	*n* (%)
Sex	
Male	12 (71)
Female	5 (29)
Modality	
PET/MRI	11 (65)
PET/CT + MRI	6 (35)
Primary site	
Genitourinary	15 (88)
Extremity	2 (12)
Histology	
Embryonal	12 (71)
Alveolar	5 (29)
Recurrence	
Local	1 (6)
Metastatic	5 (29)
Local and metastatic	2 (12)
Vitality of tumor tissue after resection	
0%	6 (35)
> 0%	5 (29)
not available	6 (35)

*CT* computed tomography, *MRI* magnetic resonance imaging, *PET* positron emission tomography

Total tumor volume was significantly reduced following chemotherapy from a mean of 11,217 voxels pre-chemotherapy to 1,625 voxels post-chemotherapy. Mean overall SUV relativized to liver SUV_mean_ was significantly reduced after chemotherapy, from 2.7 (standard deviation [SD] 1.3) to 1.1 (SD 0.5) (*P* < 0.001). In contrast, there was no significant change in mean overall ADC value following chemotherapy, from 1,378 (SD 343) [10^–6^ mm^2^/s] to 1,379 (SD 457) [10^–6^ mm^2^/s].

### Estimation of voxel-by-voxel data within groups

The proportion of voxels in the “viable tumor tissue group” decreased significantly from an average of 21.1% to 0.8% (*P* < 0.001). However, the proportion of voxels in the “intermediate tissue group” did not change significantly, from an average of 41.5% to 28.6% (*P* = 0.14). There was also a significant change in the proportion of voxels in the “possible necrosis group” from an average of 36.3% to 70.6% (*P* = 0.003).

### Gaussian mixture model clustering

**Fig. 3:**
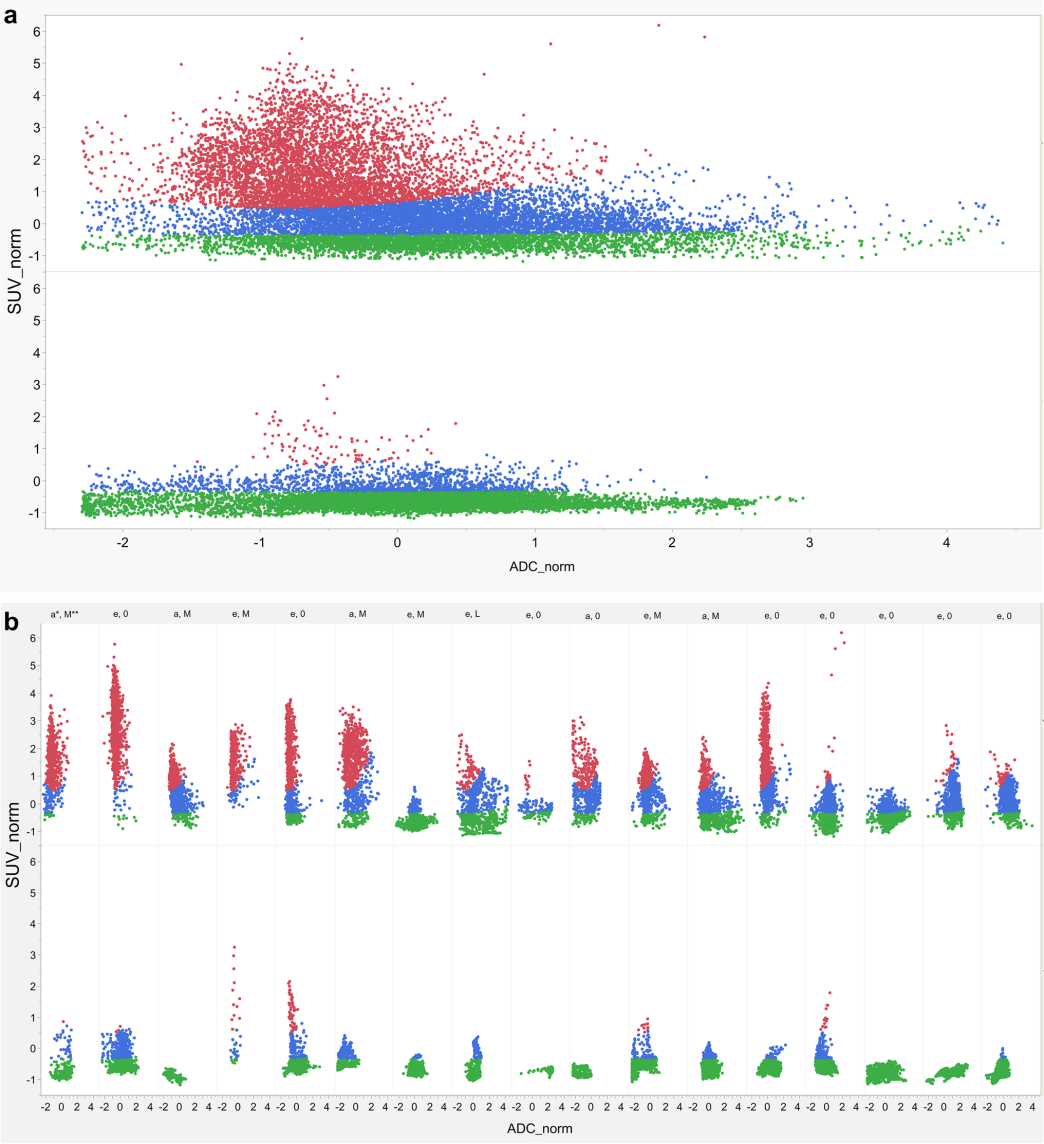
Proportion and distribution of the three clusters for all patients and separately for each patient before and after chemotherapy. cluster 1 = red, cluster 2 = blue, cluster 3 = green. **a** All patients before (*top row*) and after therapy (*bottom row*). **b** For each patient before (*top row*) and after therapy (*bottom row*). Depicted histological subtype of the rhabdomyosarcoma and status of recurrence in follow-up: * Histological subtype: *a* alveolar, *e* embryonal. ** status of recurrence: *M* metastatic, *L* local, *0* no recurrence. *ADC* apparent diffusion coefficient, *norm* normalized, *SUV* standard uptake value

**Fig. 4:**
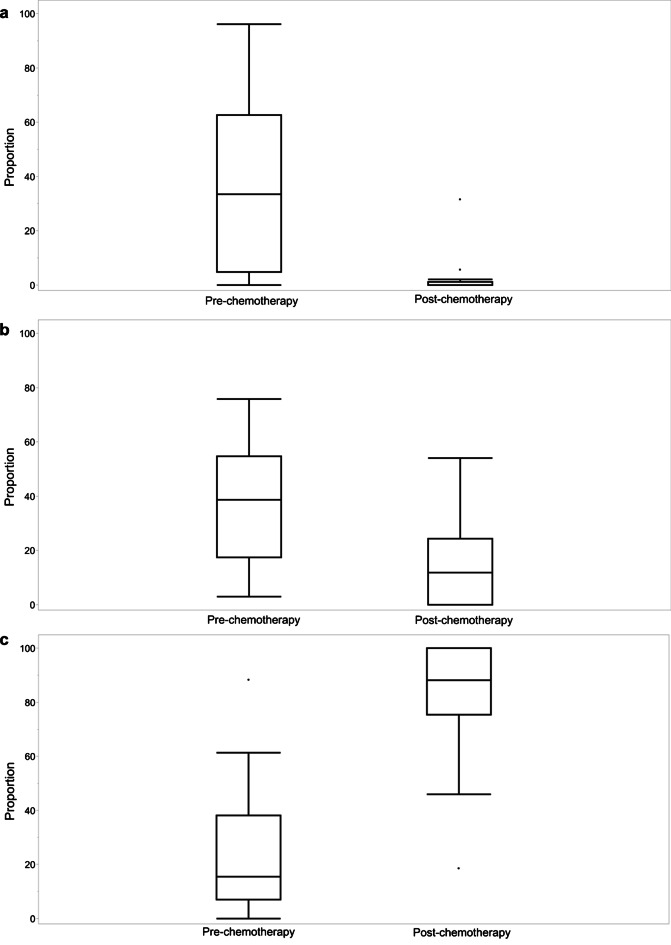
The proportion of voxels in the three clusters before and after chemotherapy: **a** cluster 1. **b** cluster 2. **c** cluster 3

Gaussian mixture model clustering using three Gaussian mixtures was successfully performed, enclosing all 34 data sets. Figure 3 shows an overview of the three clusters before and after chemotherapy and clusters separately for each patient. As depicted in Fig. 4, there was a reduction of the proportion of voxels in cluster 1 from a median of 36.4% to 0.2% (*P* < 0.001). The third cluster proportion increased following chemotherapy from 24.8% to 81.6% (*P* = 0.02). In contrast, the second cluster decreased from a median of 38.8% to 16.0% (*P* > 0.05).

**Fig. 5:**
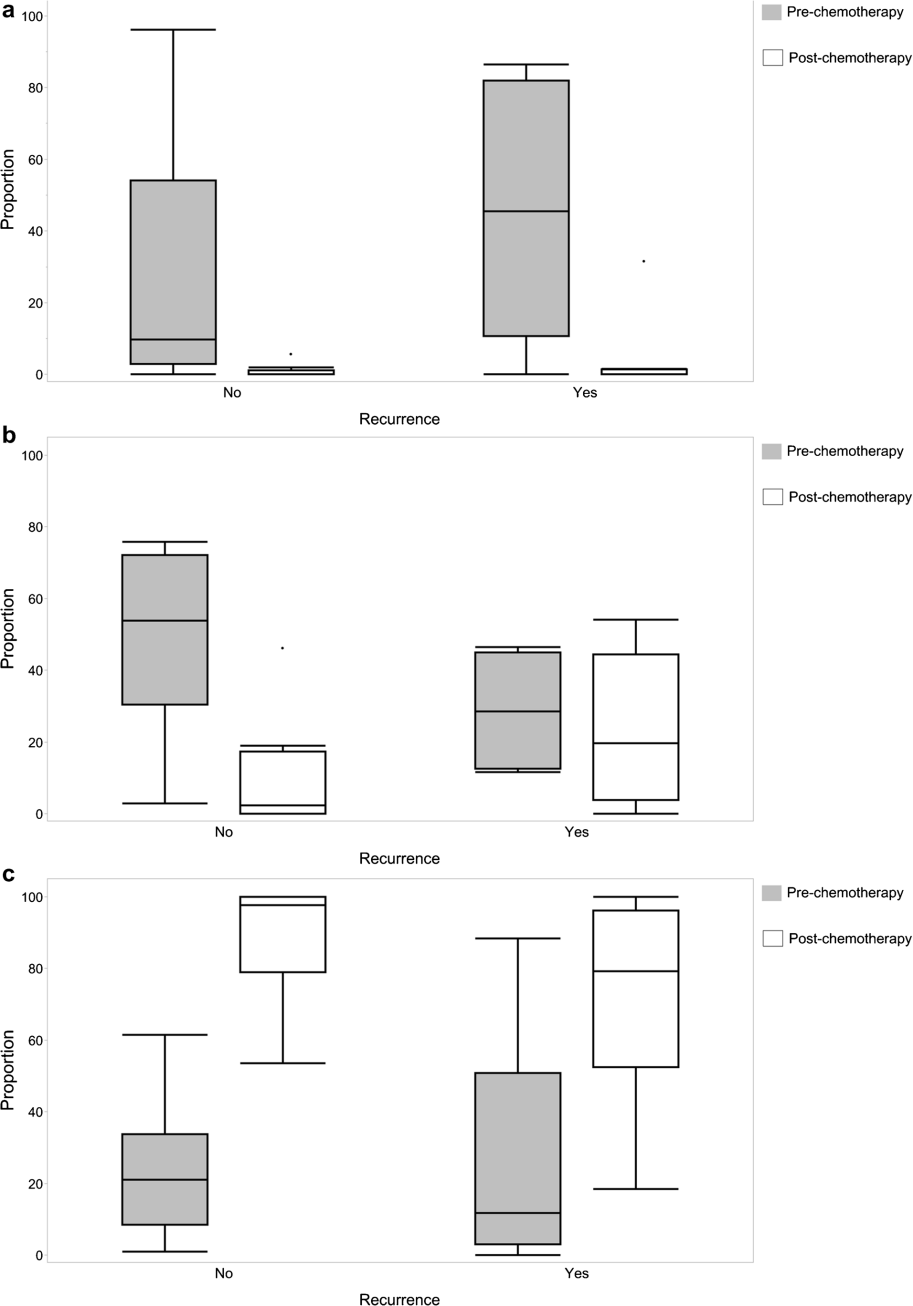
The proportion of voxels in clusters before and after chemotherapy, depending on the status of recurrence: **a** cluster 1. **b** cluster 2. **c** cluster 3

Comparing the differences in the proportions of voxels in the three different clusters before and after chemotherapy depending on whether tumor recurrence was seen on follow-up, there were significant differences in cluster 1 and cluster 3 independent of the status of recurrence. The proportion of voxels in cluster 1 reduced from 45% to 4% (*P* < 0.05) in case of recurrence, whereas it reduced from 29% to 9% (*P* < 0.05) when no recurrence occurred on follow-up. The proportion of voxels in cluster 3 increased from 23% to 69% (*P* < 0.05) post-chemotherapy in those with recurrence and increased from 27% to 73% (*P* < 0.05) when there was no recurrence. In contrast, differences in the proportions of voxels in cluster 2 before and after chemotherapy were detected depending on whether recurrence was seen on follow-up. Whereas there was no significant change when recurrence was observed, from 29% pre-chemotherapy to 23% post-chemotherapy (*P* > 0.05), a significant decrease from 48% to 10% (*P* < 0.05) was observed post-chemotherapy in children who demonstrated no tumor recurrence (Fig. 5).

## Discussion

Our initial results demonstrate the feasibility of using voxel-wise evaluation of multimodal data, including SUV in ^18^F-FDG and ADC in DWI-MRI in childhood rhabdomyosarcoma. Besides performing a volumetric characterization of tumor tissue using virtual thresholds, we demonstrated differences in proportions of tissue characteristics before and after therapy using Gaussian mixture model clustering. In other tumor entities, voxel-wise analysis of MRI, CT and hybrid imaging has been described as potent tools to improve diagnostics and therapy [22, 23]. Voxel-wise analysis using ^18^F-FDG-PET and DWI has revealed high diagnostic value in lung and breast cancer [13, 24, 25]. In this study, we demonstrate for the first time that the voxel-wise analysis of a combination of two functional/metabolic imaging parameters has potential to be an effective biomarker in children with rhabdomyosarcoma.

It is important to keep in mind that treatment stratification of malignant diseases is based on small tumor sub-volumes obtained from minimally invasive biopsies. Therefore, a precise characterization of the whole tumor volume via imaging might provide an improved target selection by identifying the most malignant parts of the tumor. Several studies demonstrate that MRI shows promise in reflecting intratumoral heterogeneity and differentiating sub-volumes, such as viable tumor tissue and necrosis [13, 26–28].

For childhood rhabdomyosarcoma, classical imaging criteria for primary tumor assessment based on tumor size are commonly used and recommended, for example by the imaging committee of the European pediatric soft tissue study group 2021 [12]. However, four of six studies failed to demonstrate a correlation between survival and anatomical response in rhabdomyosarcoma; a strong anatomical response did not correlate with higher overall survival. A correlation between anatomical response and survival could only be found when patients with progressive disease at early response measurement were included [4, 7, 8, 29–31]. In contrast, in our study, we were able to identify rhabdomyosarcoma sub-volumes on a voxel-wise basis, showing different responses to chemotherapy and varying proportions of total tumor volume, depending on whether recurrence occurred on follow-up [4, 7, 8, 28–30].

On the other hand, higher SUV_max_ at diagnosis and after induction chemotherapy was predictive for event-free survival [9]. Recently, a multicenter study has shown that patients with a metabolic tumor volume greater than 200 cm^3^ were 2.7 times more likely to have disease progression [32]. Additionally, higher ^18^F-FDG SUV_max_ and changes in ADC perfusion metrics and contrast enhancement on MRI were reported to correlate with a higher tumor grade [7, 8]. Moreover, the combination of tumor volume, high ^18^F-FDG uptake and restricted diffusion can predict outcomes. However, Harrison et al. [33] could not demonstrate a correlation between SUV_max_ or complete metabolic response with survival in rhabdomyosarcoma. No published studies investigate rhabdomyosarcoma tumor sub-volumes using our voxel-by-voxel-based approach for SUV and ADC values.

As analysis of whole tumor volumes does not predict therapy response (particularly of malignant or relevant sub-volumes of the tumor), we hypothesize an advantage of a voxel-wise, multi-parametric approach compared to conventional approaches. Our results indicate a significant difference in the proportion of voxels when comparing sub-volumes before and after chemotherapy. With both the threshold and cluster methods, the different sub-volumes in rhabdomyosarcoma seem to react differently to chemotherapy. When using thresholds to discriminate tissue, the proportion of the viable tumor tissue decreased markedly while the possible necrotic tissue increased, as expected. Thus, these groups seem to be most responsive to chemotherapy. In contrast, the proportion of the intermediate tissue only decreased moderately. Similar observations result from clustering using the multiple Gaussian distribution method. However, the proportions of the respective sub-volumes overlapped less. Moreover, the proportion of voxels in cluster 2 was different if recurrence occurred on follow-up. In cluster 2 patients with recurrence, the proportion of voxels between baseline and response was similar, whereas, in cluster 2 patients with no recurrence, a statistically significant difference was found. There were no differences regarding clusters 1 and 3 when comparing those with and without tumor recurrence. This finding is remarkable as cluster 1 contains voxels that show higher SUVs as a marker for more viable tissue. No difference in the composition of tumor volumes from sub-volumes pre- and post-chemotherapy was found based on histological subtype or treatment.

Regarding clustering results before and after chemotherapy, SUV values had greater influence than ADC values. Reinert et al. [34] previously came to a similar conclusion when investigating the possibilities of differentiating between benign and malignant lesions in neurofibromatosis using PET/MRI.

We acknowledge several limitations of our study. Firstly, the study was conducted with a relatively small patient cohort of 17, including patients with both embryonal and alveolar subtypes of rhabdomyosarcoma. However, the comparability of baseline and post-chemotherapy images led to 34 data sets. Additionally, the use of PET/MRI or even PET/CT to assess primary tumor in rhabdomyosarcoma, especially in localized disease, is unfortunately still not common practice in most sites in Europe. Therefore, a patient number of 17 comprising baseline and follow-up imaging seems acceptable.

This study was conducted as a retrospective multicenter study, including PET/MRI measurements and combined MRI with PET/CT data, which led to considerable scan variability due to images being acquired with different modalities, scanners and imaging procedures. Differences in SUV between PET/CT and PET/MRI are possible due to technical discrepancies and because there are no standardized uptake times, which may therefore differ between sites. Whereas image registration was performed automatically for PET/MRI data sets, ADC and PET data sets from PET/CT had to be registered manually. Reduction in the final image quality may occur due to motion artifacts and partial volume effects when combining MRI and PET/CT data sets acquired at different time points [35]. To minimize differences in SUV measurements due to modality and vendor, all measured SUV values were normalized to the mean SUV value of the liver. Finally, although our hypothesis is that the clusters correspond approximately to the groups: cluster 1—viable tissue, cluster 2—intermediate tissue and cluster 3— possible necrosis, we did not investigate the precise relationship between the groups according to SUV and the Gaussian-derived clusters.

To validate our results, correlation of the obtained tumor sub-volumes (viable tissue and necrosis) with a histopathological reference standard would have been desirable. In our cohort, too few patients underwent resection for a reliable statistical assessment.

## Conclusion

We have shown that voxel-based analysis and visualization of sub-volumes of rhabdomyosarcoma in children is possible. This may allow correlation with biometric, genetic and historical data for improved prediction of patient outcomes. A large prospective study should assess this further.
